# Society Meetings

**Published:** 1918-06

**Authors:** 


### SOCIETY MEETINGS

**Brief reports and announcements of meetings of societies of Western New York. and those of general scope, are requested from Secretaries. Copy should be on hand the fifteenth of the month. Full transactions will be published at cost of composition.**

---

The 21st Annual Meeting of the **Am. Gastro-Enterological Assn.** was held at Atlantic City May 6 and 7. Papers were read by Drs. R. Walter Mills of St. Louis; I. H. Levy of Syracuse; Martin E. Rehfuss of Phila.; Jacob Kaufmann of New York; Julius Friedenwall and Alexius McGlannan of Baltimore; Sidney K. Simon of New Orleans; W. A. Bastedo of New York; Franklin White of Boston; Oscar Klotz of Pittsburgh; G. E. Pfahler of Phila.; Thos. R. Brown, L. F. Barker, Baltimore; Ludwig Kast, J. W. Draper, Max Finhorn, New York; A. L. Benedict, Buffalo; G. W. McCaskey, Fort Wayne; John C. Hemmeter, Baltimore; Frank Smithies, Chicago; John Bryant, Boston, and Seale Harris, Birmingham.

---

**Rochester Academy of Medicine**, Meeting was held April 30. Officers from the Base Hospital at Rochester read the following papers: Immobilization of Fractures on the Field by Capt. Alexander S. Sims; The Barany Test in Army Examination by Lieut. B. G. Voorhees; The Present Status of Serum Therapy by Lieut. Albert D. Kaiser.

---

The Annual Meeting of the **New York Medical Society** was held in Albany, May 21st. A splendid program was given. Among the western New York State physicians who participated were: Drs. Chas. J. Hunt, Chas. W. Webb, Clifton Springs; H. U. Williams, Harry R. Trick, Ernest Watson, DeWitt H. Sherman, Irving W. Potter, F. C. Goldsborough, Lucien Howe, Carl G. Lee-Wolf, R. E. Fronczak, Buffalo; Myron B. Palmer, W. W. Brown, A. C. Snell, A. D. Kaiser, Joseph Roby, Rochester; A. A. Vander Veer, Albany; G. B. Broad, H. W. Schoeneck, J. R. Johnson, W. D. Ayer, Syracuse.

---

The regular meeting of the **Medical Society of the County of Erie** was held at the Buffalo Medical College Monday, April 15, at 8:30 p. m.

President George F. Cott called the meeting to order.

The Secretary read the minutes of the last regular meeting held on February 18, also the minutes of the Council held March 15, and of the Council held March 25, and also meeting of the Council held April 11. All of which were approved as read.

Dr. Bonnar, Chairman of the Board of Censors made a verbal report.

Dr. H. W. Cowper, Chairman of the Committee on Legislation briefly outlined the activities of his committee during the past two months and gave a prospective outline of some further work which his committee proposes to lay before the Society and which would require an appropriation in order to carry it out.

On motion of Dr. Lytle the report was accepted and the appropriation asked for granted.

Dr. Woodruff, Chairman of the Committee on Economics reported on the activities of his committee, and also outlined some work which his committee contemplates doing and which would require an appropriation to carry out.

Dr. F. Park Lewis moved that the sum of $25 be appropriated for the use of the Committee on Economics. The motion was carried.

The Secretary presented a communication and resolution from the Medical Society of the County of Schnectady, relative to the Health Insurance Law. The communication was received and filed and the Secretary directed to acknowledge the receipt.

Dr. W. F. Jacobs, Chairman of the Committee on Membership, was unable to remain at the meeting and the Secretary therefore presented the applications for membership as recommended by the Committee on Membership through Dr. Jacobs, as follows:

Dr. Willis B. Harrison, 40 Krettner St., Buffalo, N. Y.

Dr. Raymond George Laport, Emergency Hospital, Buffalo, N. Y.

Dr. Clarence Kummer, 324 Myrtle Ave., Buffalo, N. Y.

Dr. Frederick W. Palmer, Interne, Buffalo General Hospital, Buffalo, N. Y.

Dr. George M. Oppermann, 2798 Delaware Ave., Kenmore, N. Y.

All of these were duly elected to membership in the Society.

President Cott then presented Dr. Lewis Fisher of the University of Pennsylvania Hospital, Philadelphia, who gave a very interesting address on "The Value of Internal Ear Tests to the General Practitioner," illustrated by lantern slides and moving pictures, also demonstrations on living subjects.

This address was followed by a lively discussion participated in by a number of physicians present. At the conclusion a vote of thanks was tendered on motion of Dr. Buswell to Dr. Fisher for his very able, interesting and instructive address.

Dr. T. H. McKee introduced the following resolution, which was unanimously adopted:

RESOLVED, That the delegates of the Medical Society of the County of Erie and the Medical Society of the State of New York, are hereby instructed to introduce a proposition and support it by every means in their power to secure an appropriation for the purpose of public propaganda under appropriate control for the purpose of educating the public to a better understanding of the value and import of animal experimentation past and present to scientific research achievement.

A collation was tendered in the College Library at the close of the meeting.

FRANKLIN GRAM, Secretary.

---

**Buffalo Academy of Medicine,** Meeting was held May 8th. Program, Section of Medicine, ''The Clinical Basis for Treatment of Some Endocrinopathies'' by Dr. Walter Timme, New York.

---

**Elmira Academy of Medicine,** Meeting was held May 1st. Dr. Geo. M. Case read a paper on the Relation of Nasal to Eye Disturbances and Dr. Anna Stuart one on Anaesthesia.

---

**Rochester Academy of Medicine**—Meeting May 8. Program—''Etiology of Acute and Chronic Nephritis'' by Dr. Alvah S. Miller. ''The Value of Recent Laboratory Tests in Diagnosis and Treatment of Nephritis'' by Dr. C. Clyde Sutter.

---

**The Niagara District Medical Assn.** met at St. Catherines, Ont., Apr. 30, and elected officers as follows: Pres. Dr. Colbeck of Welland; V. P. Dr. Duggan of St. David's; Sec.-Treas. Dr. Cowper, Welland.

---

**The American Gastroenterologic Assn.** held its 21st annual meeting at Atlantic City, May 7 and 8.

---

**Buffalo Academy of Medicine,** Section of Obstetrics and Gynecology, May 15th. Program: Symposium: Tumors of the Ovaries by Dr. James E. King; Pathology by Dr. Chas. A. Bentz.

